# Marine Myxobacteria as a Source of Antibiotics—Comparison of Physiology, Polyketide-Type Genes and Antibiotic Production of Three New Isolates of *Enhygromyxa salina*

**DOI:** 10.3390/md8092466

**Published:** 2010-09-03

**Authors:** Till F. Schäberle, Emilie Goralski, Edith Neu, Özlem Erol, Georg Hölzl, Peter Dörmann, Gabriele Bierbaum, Gabriele M. König

**Affiliations:** 1 Institute of Pharmaceutical Biology, University of Bonn, Nussallee 6, 53115 Bonn, Germany; E-Mails: till.schaeberle@uni-bonn.de (T.F.S.); emilie.goralski@gmx.de (E.G.); e.neu@uni-bonn.de (E.N.); erdi@uni-bonn.de (Ö.E.); 2 Institute of Molecular Physiology and Biotechnology of Plants (IMBIO), University of Bonn, Karlrobert-Kreiten-Str. 13, 53115 Bonn, Germany; E-Mails: georghoelzl@uni-bonn.de (G.H.); doermann@uni-bonn.de (P.D.); 3 Institute of Medical Microbiology, Immunology and Parasitology (IMMIP), University of Bonn, Sigmund-Freud-Str. 25, 53127 Bonn, Germany; E-Mail: Gabi.Bierbaum@ukb.uni-bonn.de (G.B.)

**Keywords:** marine myxobacteria, Enhygromyxa salina, PKS-genes, antibacterial, antibiotic

## Abstract

Three myxobacterial strains, designated SWB004, SWB005 and SWB006, were obtained from beach sand samples from the Pacific Ocean and the North Sea. The strains were cultivated in salt water containing media and subjected to studies to determine their taxonomic status, the presence of genes for the biosynthesis of polyketides and antibiotic production. 16S rDNA sequence analysis revealed the type strain *Enhygromyxa salina* SHK-1^T^ as their closest homolog, displaying between 98% (SWB005) and 99% (SWB004 and SWB006) sequence similarity. All isolates were rod-shaped cells showing gliding motility and fruiting body formation as is known for myxobacteria. They required NaCl for growth, with an optimum concentration of around 2% [w/v]. The G + C-content of genomic DNA ranged from 63.0 to 67.3 mol%. Further, the strains were analyzed for their potential to produce polyketide-type structures. PCR amplified ketosynthase-like gene fragments from all three isolates enhances the assumption that these bacteria produce polyketides. SWB005 was shown to produce metabolites with prominent antibacterial activity, including activity towards methicillin resistant *Staphylococcus aureus* (MRSA) and *Staphylococcus epidermidis* (MRSE).

## 1. Introduction

Myxobacteria are Gram-negative gliding bacteria with a high G + C content, performing a complex series of cellular differentiation processes that lead to fruiting body formation. Phylogenetic studies, using 16S rDNA sequences, showed that myxobacteria are located in a homogeneous cluster within the δ-Proteobacteria. Most myxobacteria have a heterotrophic life style and the ability to lyse a variety of bacteria and fungi to obtain nutrients from lysis products. Terrestrial myxobacteria usually do not tolerate NaCl concentrations greater than 1.0%, which is clearly lower than that found in seawater with an average of 3.5% NaCl [1]. Although, sporadic reports of myxobacteria from marine environments appeared, only a few seemed to indeed be of marine origin. Myxobacterial isolates found in marine habitats being nonhalotolerant, are presumed to have arisen from myxospores of terrestrial myxobacteria which germinated under laboratory conditions. It is assumed that such spores have been transferred from the land to seawater and beach areas, thus being found in samples taken from the marine environment [2,3]. In recent times, several halophilic myxobacteria have been described and classified as novel myxobacterial genera, namely *Haliangium, Plesiocystis* and *Enhygromyxa* [4–6].

A striking characteristic of myxobacteria is their ability to produce structurally intriguing natural products with prominent biological activities, *i.e.*, 17 secondary metabolite loci are encoded in the genome of the model myxobacterium *Sorangium cellulosum* [7]. As the majority of these metabolites are polyketides, nonribosomal polypeptides, or a hybrid of both [8,9], the number of polyketide synthases (PKSs) found in these organisms is extremely high. Several myxobacterial natural products have antibiotic activity, e.g., etnangien and corallopyronin A. The first one, a macrolide antibiotic, was isolated from *Sorangium cellulosum* and its biosynthetic gene cluster was analyzed, revealing an unusual polyketide biosynthesis [10,11]. The latter was isolated from *Corallococcus coralloides* and is synthesized by a mixed PKS/nonribosomal peptide synthetase (NRPS)-system [12,13]. These organisms may use their secondary metabolites to weaken or kill their prey, a suggestion which could explain why many of them are excellent producers of bioactive secondary metabolites.

All of these features make myxobacteria an excellent source of hitherto unknown novel compounds. Terrestrial myxobacteria have been extensively investigated for their metabolite production [14]. The epothilone derivative ixabepilone is used in cancer therapy and demonstrates the potential of myxobacterial secondary metabolism [15]. Myxobacteria from unusual ecological niches, for example the marine environment, are hardly ever investigated due to the fact that their isolation and cultivation is difficult. Problems include their slow growth with the consequence that they are easily overgrown by other organisms during the isolation procedure; additionally they cannot be cultivated in rich media which results in poor cell density.

The aim of the current study is to find microorganisms which can tolerate the marine ecological pressures, e.g., the salt concentration, and to explore their potential to produce bioactive compounds, especially antibiotics. This study focuses on three strains of the taxon *Enhygromyxa salina* which we isolated and cultivated from coastal sand samples. The taxonomy was determined by 16S rDNA analysis. Cultivation experiments proved that the bacteria are physiologically adapted to the marine environment, being able to tolerate “ocean-like” NaCl concentrations of 3.5%. All strains harbor PKS genes in their genome. To date however, only strain SWB005 was shown to produce metabolites with prominent antibacterial activity, including activity towards methicillin resistant *Staphylococcus aureus* (MRSA) and *Staphylococcus epidermidis* (MRSE).

## 2. Results and Discussion

### 2.1. Morphological characterization

**Figure 1:**
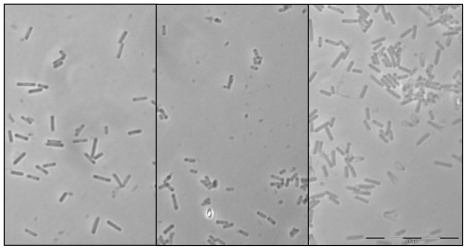
Phase-contrast micrograph of vegetative cells of the three *Enhygromyxa salina* strains cultured on VY¼ agar plates: (**a**) SWB006; (**b**) SWB005; (**c**) SWB004. (Bar = 10 μm).

During the current study, three bacterial strains (SWB004, SWB005 and SWB006) were obtained, which we were able to grow in liquid and on solid media. The vegetative cells of all three marine isolates are rod shaped with blunt rounded ends, measuring 1.2–2.6 μm in length and 0.3–0.4 μm in width when grown on agar plates (Figure 1).

All showed gliding motility and the swarms appeared as shallow sunken craters on the plates. The agar surface was sometimes shallowly etched by all three strains. The swarming colonies were colorless and formed a thin film on VY¼ agar. There were however, also some minor differences concerning the appearance of the swarming colonies of the three bacterial isolates. Thus, SWB006 was the only strain that sometimes showed deep sunken craters on the agar plates. Swarms of SWB006 and SWB004 were able to reach the rim of the plastic dish after several weeks of incubation, SWB005 however, never swarmed that far and stopped its gliding mobility after 2 cm. Radial patterns were sometimes formed by the swarms. Orange colored fruiting bodies, globular to polyhedral in shape, appeared on the surface of the swarming colonies after approximately two weeks cultivation on VY¼ agar and were barely visible without magnification. When they were crushed, spherical cells, 0.3–0.6 μm in diameter, became visible under the light microscope. In liquid culture all bacterial isolates formed cell clumps in an orange to reddish-orange shade of color. Disperse growth in liquid media was not observed. From their morphological appearance it could be concluded that these bacteria belong to the *Myxobacteriaceae*.

### 2.2. Physiological Characterization

**Figure 2:**
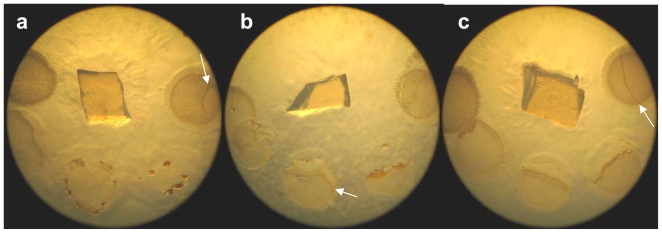
Pictures of a swarming colony on VY¼ solid medium: (**a**) SWB006, (**b**) SWB005, (**c**) SWB004. The swarming colonies are colorless. In the middle is the agar block used as inoculum. The round structures consist of living bacteria spotted on the agar. Clockwise, starting at the right hand side: *Bacillus megaterium*, *Escherichia coli*, the mixture of all cells, *Pseudomonas* spec., *Vibrio fischeri*. The white arrows indicate the border of the swarming cells. It can be seen that the swarms lyse the bacterial prey.

The phenotypic features of all three strains, along with those of the reference strain *Enhygromyxa salina* DSM 15201 are summarized in Table 1. The isolates were aerobic and able to grow on VY¼ and (very poorly on) CY⅓ agar, with SWB006 and SWB004 preferring the ionic composition of SWS, while SWB005 preferred ASW as artificial seawater (experimental section). All strains lysed living *Bacillus megaterium*, *Escherichia coli*, *Pseudomonas* spec., and *Vibrio fischeri* cells (Figure 2). Autoclaved yeast cells were only lysed in liquid cultures where clearing of the medium was observable. On agar plates, no area of clearance appeared around the colonies, meaning that the yeast cells were not decomposed. The strains did not hydrolyze cellulose, chitin, starch and tween20. On alginate, no growth at all was visible. Growth occurred at both temperatures, 19 °C and 30 °C, but not at 37 °C.

**Figure 3:**
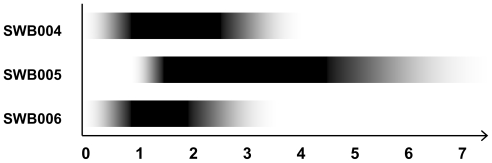
NaCl requirements of the three strains. The x-axis marks the NaCl-concentration in percent. The intensity of the black bars represents the growth characteristic (darker equates to better growth).

Marine bacteria have been defined as living in marine habitats and requiring sodium for growth [16]. Magnesium is often also needed for growth [16]. To test the marine nature of the three isolates, the influence of salinity and inorganic salts on their growth was investigated. The optimum salt concentration for growth was approximately 2% NaCl, which coincides with that for the reference strain DSM 15201. The strains however, showed some differences concerning salt requirements and tolerance (Figure 3). SWB006 showed the best growth at 1.5% NaCl and stopped growing at 4% NaCl. SWB004 had optimal growth between 1.0 and 2.5% and could tolerate up to 4.5% NaCl. Both of these strains were able to grow at low salt concentrations of 0.1% NaCl and at this low salt concentration even reached the rim of the agar dish after a long incubation time (4 weeks at 30 °C). SWB005 started growing at a salt concentration of 0.5% NaCl and was able to tolerate up to 7% NaCl. The optimal salt concentration was 2.5% NaCl. It could be hypothesized that this potential to stand higher salt concentrations than other *Enhygromyxa* strains is at the expense of growth rates. Thus, SWB005 swarms never reached the rim of an agar dish, instead they stopped swarming some centimeters away from the inoculum. The requirements concerning inorganic salts, *i.e.*, Ca^2+^, K^+^, Mg^2+^ were tested on CY⅓ agar containing 2% NaCl. In each case, the best growth was observed with all ions added to the medium. SWB005 and SWB004, however, were also able to grow when only one of these ions was supplemented. The strain SWB006 needed the supplementation of Ca^2+^ and Mg^2+^ for growth. In general, physiological differences between the strains exist, but are rather marginal. The bacterial isolates described here, possess all characteristic phenotypic features expected for marine myxobacteria, such as the requirement for NaCl and other trace elements present in seawater necessary for growth. The optimal NaCl concentration is in the range of 1–2.5%, which is characteristic of *Enhygromyxa* spp., but slightly lower than that found for other marine myxobycterial genera like *Haliangium* sp. (1–3%) and *Plesiocystis* sp. (2–3%)[4,5]. It is likely that this reflects an adaptation to seashore environments, where salt concentrations are not stable [6]. So, all three bacterial strains have properties expected for marine microorganisms and fall into the category of slightly halophilic bacteria [17].

### 2.3. Chemotaxonomy

All three bacterial isolates possessed similar fatty acid profiles, which were analyzed by GC-MS. *E. salina* strains were reported to have iso15:0, iso16:0 and iso17:0 as major fatty acids [6]. These three branched fatty acids were also the major components in SWB004, SWB005 and SWB006. Additionally, polyunsaturated fatty acid 20:4 was detected, whereas hydroxy acids were not found.

### 2.4. DNA analysis

The G + C-content of genomic DNA was determined as 63.0 mol% for SWB005, 63.7 mol% for SWB004, and 67.3 mol% for SWB006. This high G + C-content is in the range of the reported values (65–67 mol%) for other *Enhygromyxa* species [6]. The G + C-contents of *Enhygromyxa* species are thus very similar, albeit to some extent lower than those values reported for terrestrial myxobacteria. The latter have a G + C-content between 67 and 72 mol% [1].

**Figure 4:**
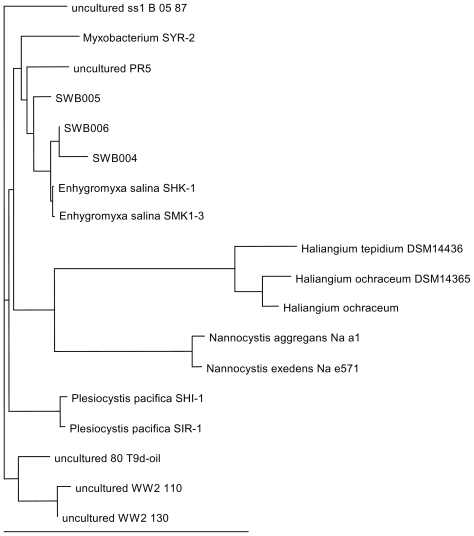
Phylogenetic dendrogram based on the 16S rDNA sequences. The bar specifies 10 nucleotide substitutions per 100 sites. The accession numbers of the sequences are: B 05 87 (EU050860), SYR-2 (AB303310), PR5 (DQ298325), SWB005 (HM769728), SWB006 (HM769729), SWB004 (HM769727), SHK-1 (AB097590), SMK1-3 (AB097591), DSM14436 (NR_024781), DSM14365 (NR_027522), *H. ochraceum* (EF108312), Na a1 (AJ233945), Na e571 (AJ233947), SHI-1 (AB016469), SIR-1 (NR_024795), 80T9d (FM242325), WW2 110 (GQ264292), WW2 130 (GQ264310).

Phylogenetic analyses were performed based on 16S rDNA sequences (sequence information obtained by PCR with the primer pair pA/pH, see experimental section). These sequences showed for all three strains 98–99% identity to the *E. salina* strains SHK-1 and SMK1–3 (Figure 4). The dendrogram is constructed on the basis of 16S rDNA sequence data of the three isolates SWB004, SWB005 and SWB006, together with sequences from representatives of formerly reported marine myxobacteria like *E. salina*, *P. pacifica*, *H. ochraceum* and *H. tepidum*. Included were also sequences from uncultured myxobacteria originating from environmental samples, and representing the best hits in a BLAST search. The three new isolates SWB004, SWB005 and SWB006 fall into one cluster with the two *E. salina* strains described by Iizuka *et al.* [6]; together with the isolates PR5 and SYR-2, originating from environmental samples, the latter one from brackish water. Our bacterial isolates thus broaden the branch of *Enhygromyxa* species within the tree of the marine myxobacteria considerably. The marine myxobacteria *Haliangium*, *Nannocystis* and *Plesiocystis* species are quite distinct from *Enhygromyxa* and each genus forms its own branch. Since only a small number of marine myxobacteria were analyzed, the phylogenetic tree in Figure 4 represents a rather preliminary picture of the relationship among these bacterial taxa and underlines the necessity to further investigate these unusual and difficult to handle bacteria. Included in the databases are, however, uncultured bacterial isolates, for which no firm confirmation of their taxonomic status is available. In these cases, it will be most important to investigate whether they are indeed part of the ascribed genus or whether they form a branch of their own.

A noteworthy fact is that when performing a BLAST search with the 16S rDNA sequence from SWB006, 37 of the first 50 hits originate from uncultured clones (Table S1). This demonstrates again the difficulties in isolating and cultivating these bacteria and the urgent need to optimize isolation and cultivation conditions.

### 2.5. The ability to produce secondary metabolites with antibacterial activity

Terrestrial myxobacteria are a rich reservoir for structurally complex secondary metabolites with various pharmacological activities. Substances with antibacterial activity being in the centre of recent investigations were for example the RNA polymerase inhibitors etnangien, corallopyronin A and ripostatin B [11,12,18]. To date, marine myxobacteria, namely *Enhygromyxa*, *Plesiocystis* and *Haliangium* species, were shown to harbor novel PKS genes, and thus have the capability for the production of new polyketides [19]. That it is reasonable to expect that marine myxobacteria are an excellent source for new bioactive small molecules was shown in 2001 by Fudou *et al.* [20]. They isolated the new antifungal antibiotic haliangicin from the marine myxobacterium *Haliangium luteum*. The activity of this metabolite is based on inhibiting the electron flow within the cytochrome b-c1 segment, and found to be superior to that of strong inhibitors, such as myxothiazol and cystothiazol.

The potential of the three strains SWB004, SWB005 and SWB006 for producing secondary metabolites was tested in two ways: (i) on the genetic level PKS genes were amplified, sequenced and compared with known sequences in the BLAST data base; (ii) the extracts of the strains were tested in agar diffusion assays for antimicrobial activity.

To analyze the sequences generated with degenerate primers for ketosynthase genes the amplificates were cloned and subsequently single clones were randomly sequenced (n = 15). For SWB004 two, for SWB005 and SWB006, in each case, three different PKS sequences were found, respectively. Even though only a small number of clones was sequenced, some sequences arose with high frequency, e.g., from the six sequences obtained for SWB004 four times a sequence showing 74% identity to a putative gene identified in *Frankia alni* str. ACN14A showed up, and twice, the highest identity (72 and 71%, respectively) was found to a putative ketosynthase gene of *Polyangium cellulosum* So0157-2-KS1. The highest identity in this screening (88%) was found between sequence #1 from SWB005 and a putative PKS gene from *Enhygromyxa* sp. SYM-1. In a recent study investigating the potential of marine bacteria for secondary metabolite biosynthesis, it was shown that sequence identities of ≥85% may ascertain which natural product is produced [21]. Taking into consideration that the two organisms are closely related, it can be assumed that the same compound may be synthesized by these two strains. The latter sequences are only distantly related to sequences from known gene clusters, and fall into a marine-specific clade of PKS-sequences [19] for which, to our knowledge, no cluster corresponding to a substance has been described so far.

The sequences found in SWB006, showed between 69 and 73% identity to PKS genes from *Streptomyces* and *Actinoplanes* species, whereas the high sequence identity between the sequences #1–4 strongly suggests that they were amplified from the same template (Table 2, Figure S1). Furthermore, only very few base substitutions (98% identity) were detectable between the sequences SWB004 #1 and SWB006 #1, which may indicate that they share the same biosynthetic pathway (Figure S2).

Extracts of all three bacterial isolates were evaluated for antimicrobial activity in agar diffusion assays. In the first tests (see experimental section), only the methanol extract of SWB005 showed activity against the Gram-positive test strain *B. megaterium* with an inhibition zone of 10 mm at a concentration of 250 μg. Testing the extracts against a broader bacterial spectrum, the methanol extract showed inhibitory activity towards methicillin resistant *Staphylococcus aureus* (MRSA), *S. epidermidis*, as well as against a coagulase-negative *Staphylococcus* sp. (Table 3). Furthermore an inhibitory activity towards MRSA was detectable in all fractions. It was found that the antibiotic activity of the SWB005 extract was strongly dependant on culture conditions, e.g., cultivation in marine broth medium revealed that SWB005 could grow in this complex medium, but the resulting extracts showed no activity.

In future work, the compounds responsible for this activity will be determined. In order to obtain sufficient biomass and production of the antibiotic compounds, cultivation conditions for this marine organism have to be further optimized, which will also allow further utilization of marine myxobacteria as an important source for natural compounds.

## 3. Experimental Section

### 3.1. Sample collection, isolation and bacterial strains

The strains SWB004, SWB005 and SWB006 were isolated from sediment samples collected in 2006–2009 from the beach area of Santa Barbara, U.S. (strain SWB005), Texel, Netherlands (SWB004) and Borkum, Germany (SWB006), respectively. Screening for myxobacteria was performed by putting the sample on an *E. coli* spot which was situated on artificial seawater (ASW, see below) agar. To inhibit the growth of fungi, cycloheximide was added to the agar. Incubation was performed at 30 °C. If lysis of the *E. coli* was observed, this sample was transferred to a new plate until an axenic culture could be obtained. The strain *Enhygromyxa salina* DSM 15201 was used in the studies as reference strain.

### 3.2. Morphological and physiological characterization

Morphology and fruiting body formation were observed on VY¼ [bakers yeast, 0.25%; CaCl_2_·2H_2_O, 1.36 g/L; vitamin B_12_, 0.5 μg/mL] agar plates incubated at 30 °C. For microscopy the cells were scraped from the agar plate and observed using a phase-contrast microscope (Olympus BX51). The physiological characterization of SWB004, SWB005 and SWB006 was in essence done as described for *E. salina* [6]. Lytic activity toward alginate, cellulose, chitin, starch and tween20 was determined on CY⅓ medium. Growth responses to different temperatures and salt concentrations were determined on VY¼ agar plates. For the determination of the cationic requirements CY⅓ + Na^+^ agar was used as the basic medium [(g/L): Bacto Casitone (Difco), 1; Bacto Yeast Extract (Difco), 0.3; NaCl, 20; Bacto Agar (Difco), 15; (pH 7.3)]. The following cations (salts, g/L) were added to the basis medium: K^+^, (KCl, 0.5); Ca^2+^, (CaCl_2_·2H_2_O, 1.0); Mg^2+^, (MgSO_4_·7H_2_O, 8.0); Ca^2+^/Mg^2+^, (CaCl_2_·2H_2_O, 1.0; MgSO_4_·7H_2_O, 8.0); and K^+^/Ca^2+^/Mg^2+^, (KCl, 0.5, CaCl_2_·2H_2_O, 1.0; MgSO_4_·7H_2_O, 8.0). As artificial seawater for the media were used SWS [(g/L): ferric citrate, 0.01; MgSO_4_·7H_2_O, 8; CaCl_2_·2H_2_O, 1; KCl, 0.5; NaHCO_3_, 0.16; H_3_BO_3_, 0.02; KBr, 0.08; SrCl_2_·6H_2_O, 0.03; Di-natrium-glycerophosphate, 0.01] and ASW [(g/L): KCl, 0.66; KBr, 0.1; NaCl, 23.48; MgSO_4_·7H_2_O, 10.61; CaCl_2_·2H_2_O, 1.47; SrCl_2_·6H_2_O, 0.04; NaSO_4_, 3.92; NaHCO_3_, 0.19; H_3_BO_3_, 0.03], respectively. The complex marine broth medium was obtained from Difco.

### 3.3. Chemotaxonomy

Fatty acid composition was analyzed using GC/MS. Therefore the cell pellet was vacuum-dried and dissolved in 1 mL methanolic HCl (1N) to generate fatty acid methyl esters (FAMEs). After 20–30 min incubation in an 80 °C water bath 1 mL 0.9% NaCl and 1 mL hexane were added. After solvent extraction, the organic phase was transferred into a new vial and dried. To test if there are any hydroxylated fatty acids present in the sample, a silylation step followed, which enabled a better detection of these FAMEs. Therefore 600 μL MSTFA (*N*-Methyl-*N*-(trimethylsilyl)-2,2,2-trifluoracetamid) was added to the sample and incubation was performed 20–30 min in an 80 °C water bath. Subsequently the samples were dried and resolved in 50 μl hexane. Fatty acid methyl esters were separated by GC-MS on an Agilent HP 6890 plus GC with mass selective detector 5973inert (Agilent Technologies, Böblingen, Germany), equipped with a 30 m HP-5MS 19091S-433 column (Agilent) using a temperature gradient starting at 150 °C, increased up to 280 °C at 10 °C/min (holding time 10.5 min), and decreased to 150 °C at 20 °C/min.

### 3.4. DNA analysis

Genomic DNA was isolated with the Promega DNA purification kit according to the manufacturer’s instructions. For the 16S rDNA analysis PCRs were performed with the primer pair pA (5′AGAGTTTGATCCTGGCTCAG3′) and pH (5′AAGGAGGTGATCCAGCCCCA3′). The resulting DNA fragments were gel-purified and subsequently sequenced by GATC, Constance (Germany). For the amplification of the ketosynthase part of the PKS genes the degenerated primer pair KSup (5′MGNGARGCNNWNSMNATGGAYCCNCARCANMG3′) and KSdn (5′GGRTCNCCNARNSWNGTNCCNGTNCCRTG3′) was used. The obtained PCR fragments were gel-purified and subsequently ligated into the cloning vector pGEM-T (Promega). Calciumchloride competent *E. coli* XL1 Blue cells were transformed with the resulting plasmids and plated on ampicillin containing selection agar. Clones were randomly picked and plasmids were isolated using the Pure Yield Miniprep System kit (Promega). The inserts of these plasmids were sequenced by GATC, Constance (Germany). The sequences were analysed using nucleotide BLAST and ClustalW [22,23]. Phylogenetic trees were constructed with ClustalW.

The DNA G + C contents were determined using HPLC [24]. The values are given in % and are the means of three independent analyses of the same DNA sample. The analysis was performed by the Belgium Co-ordinated Collections of Micro-organisms (BCCM^TM^, Gent, Belgium).

The accession numbers of the 16S rDNA sequence data for the strains SWB004, SWB005 and SWB006 are HM769727, HM769728 and HM769729, respectively.

### 3.5. Agar diffusion assay

Cultivation of the strains was performed in 5-l-Erlenmeyer flasks, each containing 1 L of VY¼ medium with 2% amberlite XAD-16 (Fluka, Germany). The flasks were inoculated with a preculture from sheared agar plates and shaken on a rotary shaker (140 rpm) at 30 °C for 14 days. At the end of the cultivation, bacterial cells and adsorber resin were separated from the culture broth by centrifugation and extracted with acetone (1.5 L).

After removal of the solvent, the residue was suspended in methanol 60% (50 mL) and extracted four times with dichloromethane (DCM; 60 mL). The DCM layers were combined and dried. Separation of this extract into four fractions was done by vacuum liquid column chromatography over Polygoprep 60-50 RP (Macherey-Nagel) by consecutively employing DCM, ethylacetate, acetone and methanol. 50 μL of the resulting fractions (concentration: 5 mg/mL) were dropped on a filter disc and put on the agar plates with the test organisms (namely *Bacillus megaterium*, *Chlorella fusca*, *Escherichia coli*, *Eurotium rubrum*, *Microbotryum violaceum* and *Mycotypha microspora*). For the following agar diffusion assay with pathogenic bacteria, the suspension of bacterial strains was floated on Müller-Hinton-Agar. Filter discs with 40 μL extract (5 mg/mL) were put onto the agar plates and incubation was performed over night at 34 °C. The test strains were from the collection of the Institute of Medical Microbiology, Immunology and Parasitology (IMMIP).

## Figures and Tables

**Table 1:** Phenotypic characteristics of the new isolates compared with *E. salina* DSM15201.

	SWB005	SWB006	SWB004	DSM 15201
Colony color (on VY1/4)	colorless	colorless	colorless	colorless
**Rod cell**#:				
Diameter (μm)	ca. 0.35	ca. 0.35	ca. 0.35	0.5–0.8 *
Length (μm)	1.2–1.8	1.3–2.6	1.2–2.6	1.5–7.0 *
**Myxospore:**				
Shape	spherical	spherical	spherical	spherical *
Diameter (μm)	0.3–0.5	0.3–0.6	0.3–0.6	0.5–0.7 *
**Growth at:**				
19 °C	yes	yes	yes	yes *
30 °C	yes	yes	yes	yes
37 °C	no	no	no	no *
**Growth salinity (%NaCl):**				
Range	1.0–7.0	0.1–3.5	0.1–4.0	0.1–4.0 *
Optimum	1.5–4.5	1.0–2.0	1.0–2.5	1.0–2.0 *
Cation requirement	Ca^2+^ or Mg^2+^ or K^+^	Ca^2+^ and Mg^2+^	Ca^2+^ or Mg^2+^ or K^+^	Ca^2+^ or Mg^2+^ or K^+^
Oxidase	yes	yes	yes	yes
Catalase	yes	yes	yes	yes
**Hydrolysis of:**				
Alginate	no growth	no growth	no growth	no
Cellulose	no	no	no	no
Chitin	no	no	no	no
Starch	no	no	no	no
Tween 20	no	no	no	no growth
Yeast cells (autoclaved)	weak	yes	yes	yes
*E. coli* cells (alive)	yes	yes	yes	yes
G + C content (mol%)	63.0	67.3	63.7	65–67 *
Preferred artificial seawater	ASW	SWS	SWS	SWS

* Data were obtained from Iizuka *et al.* [6];

# The fact that the dimensions of the cells were a little bit smaller compared to the original publication can be due to the fact that these results were obtained from cells and spores grown on agar plates and not in liquid medium.

**Table 2:** Sequences of the amplified PKS-like fragments of the three *Enhygromyxa salina* strains and their closest homologs. The results of the nucleotide BLAST search are given. The genes with the highest identity and the corresponding strains are named.

Sequence	Identity	Strain and (putative) function	Accession number
SWB004 #1	512/690 (74%)	*Frankia alni* str. ACN14A, putative 6-methylsalicylic acid synthase	CT573213.2
SWB004 #2	512/690 (74%)	*Frankia alni* str. ACN14A, putative 6-methylsalicylic acid synthase	CT573213.2
SWB004 #3	512/690 (74%)	*Frankia alni* str. ACN14A, putative 6-methylsalicylic acid synthase	CT573213.2
SWB004 #4	519/714 (72%)	*Polyangium cellulosum* So0157-2-KS1 beta-ketoacyl synthase gene	DQ359910.1
SWB004 #5	512/714 (71%)	*Polyangium cellulosum* So0157-2-KS1 beta-ketoacyl synthase gene	DQ359910.1
SWB004 #6	512/690 (74%)	*Frankia alni* str. ACN14A, putative 6-methylsalicylic acid synthase	CT573213.2
SWB005 #1	567/638 (88%)	*Enhygromyxa* sp. SYM-1 gene for polyketide synthase	AB376534.1
SWB005 #2	530/712 (74%)	*Streptomyces abikoensis* gene for PKS	AB430936.1
SWB005 #3	541/706 (76%)	*Polyangium cellulosum* So ce26-KS7 beta-ketoacyl synthase gene	DQ359879.1
SWB005 #4	530/712 (74%)	*Streptomyces abikoensis* gene for PKS	AB430936.1
SWB006 #1	367/499 (73%)	*Streptomyces* sp. NRRL 11266 tetronomycin gene cluster	AB193609.1
SWB006 #2	449/607 (73%)	*Streptomyces* sp. ID05-A0343, gene for PKS	AB431909.1
SWB006 #3	350/474 (73%)	*Actinoplanes* sp. ID05-A0405, gene for PKS	AB432011.1
SWB006 #4	511/694 (73%)	*Streptomyces* sp. ID05-A0343, gene for PKS	AB431909.1
SWB006 #5	288/412 (69%)	*Streptomyces noursei* ATCC 11455, nystatin biosynthetic gene cluster	AF263912.1

**Table 3:** Inhibition zones of the SWB005 extracts against bacterial strains in agar diffusion assay. The inhibition zone is given in millimeters (mm), the filter discs were 6 mm in diameter. Concentration of the extracts was 5 mg/mL; 40 μL were spotted on the discs.

Strain\Extract	Dichloromethane	Ethylacetate	Acetone	Methanol
MRSA LT1334	13	14	11	8
MRSA LT1338	10	10	8	8
MRSE LT1324	-	-	m.i.	11
CNS I-10925	-	-	-	m.i.
*Escherichia coli* I-11276b	-	-	-	-
*Escherichia coli* O-19592	-	-	-	-
*Klebsiella pneumoniae* I-10910	-	-	-	-
*Pseudomonas aeruginosa* 4991	-	-	-	-
*Pseudomonas aeruginosa* I-10968	-	-	-	-
*Citrobacter freundii* I-11090	-	-	-	-
*Staphylococcus aureus* I-11574	-	-	m.i.	9
*Staphylococcus aureus* 5185	-	-	m.i.	9
*Stenotrophomonas maltophilia* O-16451	-	-	-	-
*Stenotrophomonas maltophilia* I-10717	-	-	-	m.i.
*Enterococcus* I-11305b	-	-	-	9
*Enterococcus* I-11054	-	-	m.i.	9
*Staphylococcus simulans* 22	-	-	m.i.	21
*Micrococcus luteus* ATCC 4658	-	-	10	18
*Staphylococcus epidermidis* 25	-	-	-	13
*Staphylococcus aureus* SG511	-	-	9	14

MRSA: methicillin resistant *Staphylococcus aureus*; MRSE: methicillin resistant *Staphylococcus epidermidis*; CNS: coagulase-negative *Staphylococcus* sp.; -: not active; m.i.: minimal inhibition.
